# Surge of Peripheral Arginine Vasopressin in a Rat Model of Birth Asphyxia

**DOI:** 10.3389/fncel.2018.00002

**Published:** 2018-01-19

**Authors:** Milla Summanen, Susanne Bäck, Juha Voipio, Kai Kaila

**Affiliations:** ^1^Department of Biosciences, University of Helsinki, Helsinki, Finland; ^2^Neuroscience Center and HiLife, University of Helsinki, Helsinki, Finland

**Keywords:** arginine vasopressin (AVP), copeptin, birth asphyxia, hypothalamic-pituitary axis (HPA axis), blood gases, base deficit, perinatal, neonatal

## Abstract

Mammalian birth is accompanied by a period of obligatory asphyxia, which consists of hypoxia (drop in blood O_2_ levels) and hypercapnia (elevation of blood CO_2_ levels). Prolonged, complicated birth can extend the asphyxic period, leading to a pathophysiological situation, and in humans, to the diagnosis of clinical birth asphyxia, the main cause of hypoxic-ischemic encephalopathy (HIE). The neuroendocrine component of birth asphyxia, in particular the increase in circulating levels of arginine vasopressin (AVP), has been extensively studied in humans. Here we show for the first time that normal rat birth is also accompanied by an AVP surge, and that the fetal AVP surge is further enhanced in a model of birth asphyxia, based on exposing 6-day old rat pups to a gas mixture containing 4% O_2_ and 20% CO_2_ for 45 min. Instead of AVP, which is highly unstable with a short plasma half-life, we measured the levels of copeptin, the C-terminal part of prepro-AVP that is biochemically much more stable. In our animal model, the bulk of AVP/copeptin release occurred at the beginning of asphyxia (mean 7.8 nM after 15 min of asphyxia), but some release was still ongoing even 90 min after the end of the 45 min experimental asphyxia (mean 1.2 nM). Notably, the highest copeptin levels were measured after hypoxia alone (mean 14.1 nM at 45 min), whereas copeptin levels were low during hypercapnia alone (mean 2.7 nM at 45 min), indicating that the hypoxia component of asphyxia is responsible for the increase in AVP/copeptin release. Alternating the O_2_ level between 5 and 9% (CO_2_ at 20%) with 5 min intervals to mimic intermittent asphyxia during prolonged labor resulted in a slower but quantitatively similar rise in copeptin (peak of 8.3 nM at 30 min). Finally, we demonstrate that our rat model satisfies the standard acid-base criteria for birth asphyxia diagnosis, namely a drop in blood pH below 7.0 and the formation of a negative base excess exceeding −11.2 mmol/l. The mechanistic insights from our work validate the use of the present rodent model in preclinical work on birth asphyxia.

## Introduction

In all mammalian species, the shift during parturition from maternal-fetal umbilical respiratory gas exchange to the activation of fetal lungs is associated with a transient period of asphyxia in the neonate. By definition, asphyxia implies a fall in blood O_2_ (hypoxia) that is associated with an elevation of CO_2_ (hypercapnia). This period of obligatory, non-pathophysiological asphyxia is an essential part of the “stress of birth” (Van Woudenberg et al., 2012; Evers and Wellman, 2016), which is beneficial in that it activates the hypothalamic-pituitary axis (the HPA axis) as well as the sympathetic nervous system. This, in turn, triggers a wide range of cardiovascular and pulmonary responses facilitating the adaptation of the newborn individual to extra-uterine life (Lagercrantz and Slotkin, 1986; Van Woudenberg et al., 2012; Lagercrantz, 2016). However, a prolonged period of birth asphyxia is harmful, causing dysfunction and damage with lifelong consequences in organ systems with a high and predominantly aerobic energy metabolism, such as the brain (Painter, 1995; Fattuoni et al., 2015; Ahearne et al., 2016).

In humans, various kinds of birth complications, such as umbilical occlusion, prolonged labor, and delay in neonatal lung function, can cause a pathophysiological state diagnosed as clinical birth asphyxia, which is one of the leading causes of neonatal death worldwide (Lawn et al., 2010; Lee et al., 2013). While a lack of O_2_ has deleterious effects on a number of organ systems, it is obvious that the neonatal human brain is particularly sensitive to asphyxia-induced damage, known as hypoxic-ischemic encephalopathy (HIE). Thus, survivors of moderate to severe HIE show a high incidence of neurological and psychiatric disorders later in life, including cerebral palsy, mental retardation, mood disturbances, schizophrenia, and epilepsy (Morales et al., 2011; Ahearne et al., 2016). In order to understand the physiological mechanisms causing HIE, and to develop therapies for the treatment of HIE, a wide variety of animal models ranging from standard laboratory rodents to large mammals such as piglets and sheep have been used to explore the mechanisms, as well as short- and long-term consequences, of birth asphyxia (Raff et al., 1991; Painter, 1995; Vannucci and Vannucci, 1997; Johnston et al., 2005; Fattuoni et al., 2015; Mallard and Vexler, 2015).

Most of the basic and clinical research on birth asphyxia has focused on the hypoxia-induced defects in brain energy metabolism (Tusor and Edwards, 2014), and on the consequences thereof. However, the period of obligatory birth asphyxia related to normal birth triggers massive neuroendocrine activation, leading to an increase in the circulating levels of various stress hormones such as arginine vasopressin (AVP) and catecholamines (Chard et al., 1971; Lagercrantz and Slotkin, 1986; Wellmann et al., 2010). Even after normal vaginal birth, the amount of AVP released into the circulation in the human neonate reaches a level which is higher than under any physiological or pathophysiological condition later in life (Chard et al., 1971; Wellmann et al., 2010; Evers and Wellman, 2016). Notably, the surge in AVP secretion is further enhanced by pathophysiological (clinical) birth asphyxia (Schlapbach et al., 2011; Evers and Wellman, 2016; Summanen et al., 2017).

AVP is mainly produced in the magnocellular neurons of the paraventricular nucleus (PVN) and the supraoptic nucleus (SON) of the hypothalamus, and released into the circulation from the posterior pituitary (Koshimizu et al., 2012; Evers and Wellman, 2016). AVP is highly unstable, with a plasma half-life of only a couple of minutes, but the C-terminal part of preproAVP, copeptin, that is released in an equimolar ratio to AVP, is biochemically more stable, and has a plasma half-life of around 30 min (Morgenthaler et al., 2006; L'Abate et al., 2013; Evers and Wellman, 2016). The relatively long half-life of copeptin provides a useful time-integrated signal of the net release of AVP. Thus, copeptin is an ideal surrogate marker of peripheral AVP release into the circulation.

Despite the relevance of AVP release for the diagnosis, mechanisms, and therapies of birth asphyxia, and the prevalence of rodent models of birth asphyxia, to our knowledge, there are so far no studies investigating the role of AVP in birth asphyxia in laboratory rodents (but see reference Spoljaric et al., 2017). Here we show for the first time that normal birth is accompanied by an AVP surge also in the rat, and that in a novel rat model of birth asphyxia, where 6-day old rat pups are exposed to a 45 min period of steady or intermittent asphyxia, AVP release as measured using copeptin can be reliably evoked. Furthermore, we demonstrate that this model satisfies the standard diagnostic criteria (Azzopardi et al., 2009; Schlapbach et al., 2011; Summanen et al., 2017) of human birth asphyxia based on blood pH and negative base excess (BE). Our results provide important information on the kinetics of AVP/copeptin release during asphyxia, and validate the use of the current rodent model in preclinical work on birth asphyxia.

## Materials and methods

### Animals

The experiments were performed on postnatal day (P) 0 female or male, and P6 male Wistar rat pups. The P0 rats consisted of two cohorts with one of them born normally, and the other one taken just before birth by cesarean section at embryonic day (E) 21.5 (see below). Both males and females were used at P0 since we prioritized fast blood sample collection, and sexing newborn animals would have delayed this process to a degree that would have made the basic design and rationale of these experiments meaningless. All procedures were approved by the National Animal Ethics Committee of Finland and the local animal ethics committee of the University of Helsinki.

### Perinatal blood sampling

Rat embryo blood samples were collected at E21.5 from timed-pregnant rat dams. The dams were anesthesized with 4.5% isoflurane, and anesthesia was maintained with 2.0–2.3% isoflurane during cesarean section, after which the dam was decapitated. After cesarean section, the embryos were immediately decapitated and trunk blood was collected into EDTA-coated tubes and placed on ice. Protease inhibitors (Complete, Roche) were added and the samples were centrifuged at 1,300 g, 4°C for 10 min. The plasma taken from all embryos (9–15 pups/litter) in a given litter was combined into one sample and stored at −80°C until analysis. Blood samples collected at birth were taken within 2 min after normal delivery, and samples from four pups from the same litter were combined into one sample. Samples collected at 5–6 h postnatal were from individual animals that experienced normal birth.

### Experimental asphyxia and blood sample collection

All treatments were done in a closed experimental chamber at a temperature of 33°C (chamber volume ~1.5 L). The gas mixtures (including room air) were humidified and pre-warmed, and applied into the chamber at a constant flow rate of 1.5 L min^−1^. Pups were put into the closed chamber 15 min before applying a gas mixture with compositions given below.

To study the time-dependent changes in copeptin levels induced by experimental asphyxia, a gas mixture containing 4% O_2_, 20% CO_2_ and 76% N_2_ (4% O_2_/20% CO_2_ asphyxia) was applied for 5, 10, 15, or 45 min. Trunk blood samples were collected immediately after the treatment, or, alternatively, after 45 min of asphyxia followed by exposure to room air for 30, 60, or 90 min. Control animals were treated with room air, and samples were collected at the same time points, except for 5 and 10 min. In order to compare the copeptin levels between the 4% O_2_/20% CO_2_ asphyxia model described above, and the asphyxia model used in our previous work (Helmy et al., 2011, 2012), male P6 rat pups were also exposed to 9% O_2_, 20% CO_2_, and 71% N_2_ (9% O_2_/20% CO_2_ asphyxia) for a period of 60 min. Trunk blood samples were collected at 15 min of asphyxia, and at 30 min post-asphyxia. To check whether experimental asphyxia can evoke AVP/copeptin release already at P0, pups were exposed to 15 min of 4% O_2_/20% CO_2_ asphyxia or room air (controls) 5–6 h after birth, and trunk blood samples were collected.

A model of intermittent asphyxia was developed to more accurately simulate prolonged, complicated labor. In this model, male P6 rat pups were exposed to alternating, 5 min periods of 9% O_2_/20% CO_2_ asphyxia and 5% O_2_/20% CO_2_ asphyxia (5% O_2_, 20% CO_2_, and 75% N_2_), for a total of 45 min. Trunk blood samples were collected after 10, 20, 30, and 45 min of asphyxia, as well as after 45 min of asphyxia followed by exposure to room air for 30, 60, or 120 min. Finally, the effects of hypoxia only (4% O_2_ in N_2_) and hypercapnia only (20% CO_2_, 20% O_2_, and 60% N_2_) were studied by applying these gas mixtures to the rats for 15 and 45 min.

Samples of trunk blood were collected into EDTA-coated tubes and placed on ice. Protease inhibitors (complete, Roche) were added, and the samples were centrifuged at 1,300 g, 4°C for 10 min. The plasma was stored at −80°C until analysis. To avoid degradation of copeptin during storage, all samples were analyzed on the ELISA/AlphaLISA within 2 months after the date of collection. Rats from at least two litters were used for each experimental group at P6, and all samples were from individual rat pups (thus, in contrast to the P0 animals, no combined samples had to be used).

### Blood gas analyses

Blood gas analyses were performed with a standard clinical GEM Premier 4000 (Instrumentation Laboratory, Bedford, MA, USA) blood gas analyzer. The blood was collected from the trunk of the animal into a 80 μl heparin-coated capillary immediately after decapitation, and analyzed within 5 min after collection. The “mixed venous” micro-protocol was used for the analysis due to the sample type and obtainable volume. This protocol has a pH cut-off of 6.8, and a pCO_2_ cut-off of 20 kPa, which prevent the observation of lower and higher values, respectively. Bicarbonate concentration ([HCO3]) was calculated according to the following formula:

$$[{\text{HCO}}_{3}]={\text{pCO}}_{2}\times 7.502\times 10^{\text{pH}-7.608}$$

where numerical values of the partial pressure of CO_2_ (pCO_2_) are substituted without their unit of kPa, and the obtained [HCO_3_] values are in mM. The bicarbonate concentration was used to calculate the base excess (BE, in mM):

$$\begin{array}{l} \text{BE}=\left[{\text{HCO}}_{3}\right]-24.8+16.2\times \left(\text{pH}-7.4\right) \end{array}$$

In four out of five pups exposed to 4% O_2_/20% CO_2_ for 15 min, the pH was below 6.8 (in one pup the pH was 6.85), and in all five pups the pCO_2_ was above 20 kPa. With these cut-off values of pH and pCO_2_ the above equations yield a BE of −11.2 mM, which, however, is not a cut-off value for BE (a lower value was calculated for one pup exposed to hypoxia; see **Figure 5C**).

### Copeptin ELISA

Copeptin concentrations in rat plasma were measured using a sandwich ELISA (Summanen et al., 2017). Nunc Maxisorp plates (Thermo Scientific) were coated with 1 μg of sheep anti-copeptin (gift from Thermo Fisher Scientific) per well in 100 μl of PBS overnight at 4°C. The next day, wells were blocked for 1 h RT shaking with 200 μl of 4% skimmed milk in PBS (PBS-M) per well. A serial dilution (0–40 ng/ml) of rat copeptin (Bachem) was made in 2% PBS-M and used as a standard on every ELISA plate. Rat plasma samples were diluted 2.5 times in 2% PBS-M and 100 μl of standards and samples were pipetted to the plate in duplicate and incubated with shaking for 2 h at RT. Next, wells were washed 3 times with 200 μl of PBS per well and bound copeptin was detected with a 1:1,000 dilution of goat anti-copeptin (Santa Cruz Biotechnology) in 2% PBS-M (1 h at RT shaking, 100 μl/well). After washing the plate 6 times with PBS, the plate was incubated for 1 h at RT shaking with a 1:5,000 dilution of mouse anti-goat horse radish peroxidase (HRP)-conjugated secondary antibody (Abcam) in 2% PBS-M. Antibody binding was visualized with o-phenylenediamine dihydrochloride (OPD; SigmaFast tablets, Sigma) and the plate was scanned at 450 nm with a plate reader (Biorad) 30 min after OPD addition.

### Copeptin AlphaLISA

To improve the sensitivity of the copeptin assays, an alternative method using the same copeptin antibodies was developed based on AlphaLISA technology (PerkinElmer). The goat anti-copeptin antibody (SantaCruz Biotechnology) was conjugated to AlphaLISA acceptor beads according to the manufacturer's instructions. The sheep anti-copeptin antibody was biotinylated with EZ-LinkTM Sulfo-NHS-SS-Biotin (Thermo Scientific). A serial dilution of rat copeptin (0–160 ng/ml) in adult rat plasma devoid of copeptin was used as a standard.

The assay was performed in triplicate in 384-well Alpha plates. First, 5 μl of blank plasma, standard or sample was pipetted to the plate, and 10 μl of goat anti-copeptin conjugated AlphaLISA acceptor beads at a final concentration of 20 μg/ml were added to each well. After 1 h incubation, 10 μl of biotinylated sheep anti-copeptin at a final concentration of 5 nM was added to each well, and the plate was incubated for 1 h. Finally, 25 μl of AlphaLISA Streptavidin donor beads at a final concentration of 40 μg/ml was added to each well, and after incubating for 1 h, the plate was read with an EnSpire multimode plate reader (PerkinElmer). The resulting AlphaLISA signal was analyzed using a 4-parameter logistic regression curve fit on GraphPad Prism. The lowest detection limit (LDL), calculated as 3 times the standard deviation of the blank mean, was 0.2 nM. The average coefficient of variation (CV) from the three replicates per sample was 3.5%. The ELISA and AlphaLISA methods gave identical results for samples collected in the same way (see Supplementary Figure 1).

### Statistical analysis

Data are given as mean ± SEM. The statistical analyses were performed with GraphPad Prism 6. The copeptin concentrations between treatment groups were compared using the Mann–Whitney *U*-test or the Kruskal–Wallis test. The blood gas parameters between groups were compared with the Kruskal–Wallis test.

## Results

### Increase in serum copeptin at birth in the rat

**Figure 1:**
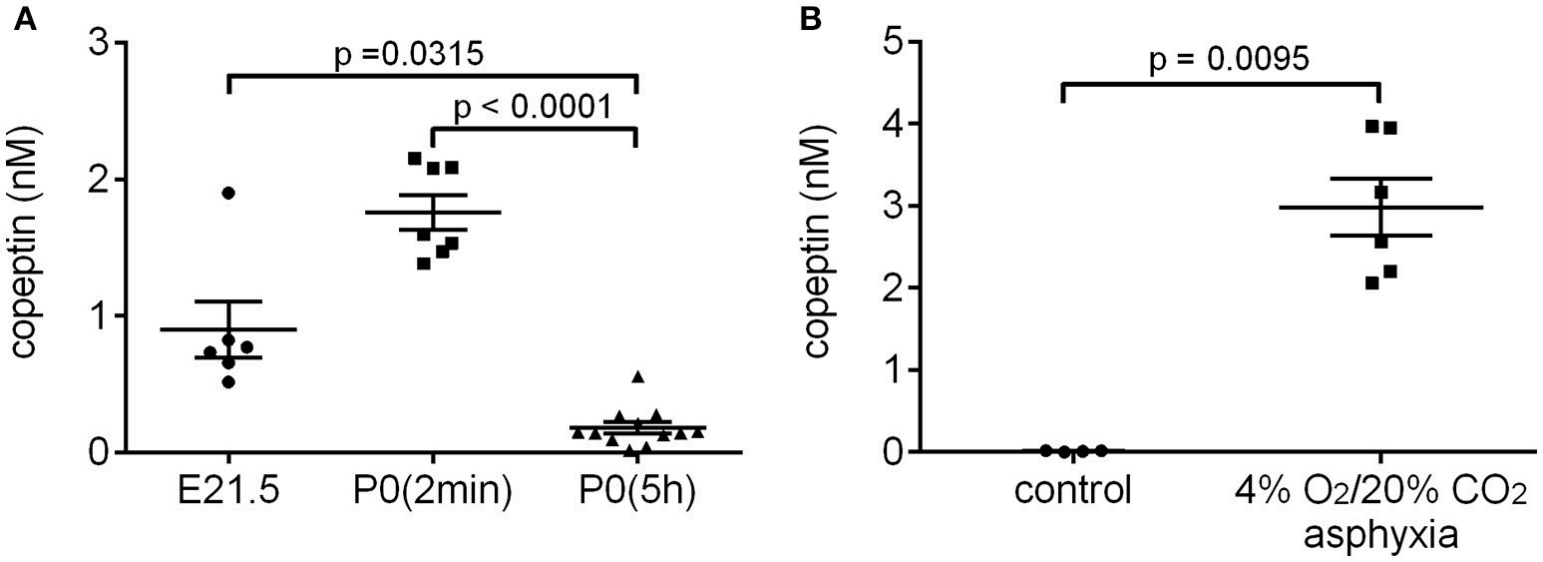
Normal birth **(A)** and experimental asphyxia **(B)** increase copeptin levels in P0 rat pups. **(A)** E21.5 samples were collected after cesarean section of the dam, and blood samples taken from all pups in a given litter were pooled into one sample for analysis. The P0(2 min) samples were collected within 2 min after normal birth, and blood from 4 pups from the same litter was pooled for each sample. P0(5 h) samples were collected 5–6 h after normal birth, and blood from each pup was analyzed separately. **(B)** Copeptin levels in P0 pups exposed to 4% O_2_/20% CO_2_ asphyxia for 15 min, and in controls breathing room air. Blood from each pup was analyzed separately. The mean ± SEM is shown for each time point, *p*-values from the Kruskal–Wallis test **(A)** or Mann–Whitney *U*-test **(B)** are shown when the difference was statistically significant.

Several studies have shown that AVP/copeptin levels are increased after normal vaginal birth in human neonates (Chard et al., 1971; Wellmann et al., 2010), whereas neonates born by elective cesarean section generally have very low serum copeptin levels (Wellmann et al., 2010). Rather surprisingly, we have not been able to find any literature on the likely birth-induced AVP response in standard laboratory rodents. Therefore, we collected trunk blood samples from rat pups after cesarean section just before birth (E21.5), within 2 min after birth [P0(2 min)], and 5 h after birth [P0(5 h)]. The copeptin levels showed a significant increase in the P0(2 min) pups (mean 1.8 ± 0.1 nM), and had returned to baseline levels 5 h after birth (Figure 1A). These data indicate that normal birth increases peripheral AVP/copeptin release in rats as well as in humans, pointing to the presence of the evolutionary highly conserved birth-associated protective endocrine response also in laboratory rodents (see section Introduction). This conclusion gained further support from the fact that a 15 min period of experimental asphyxia induced a pronounced increase in plasma copeptin in all P0(5 h) rat pups tested (Figure 1B).

### Experimental asphyxia increases peripheral copeptin levels

**Figure 2:**
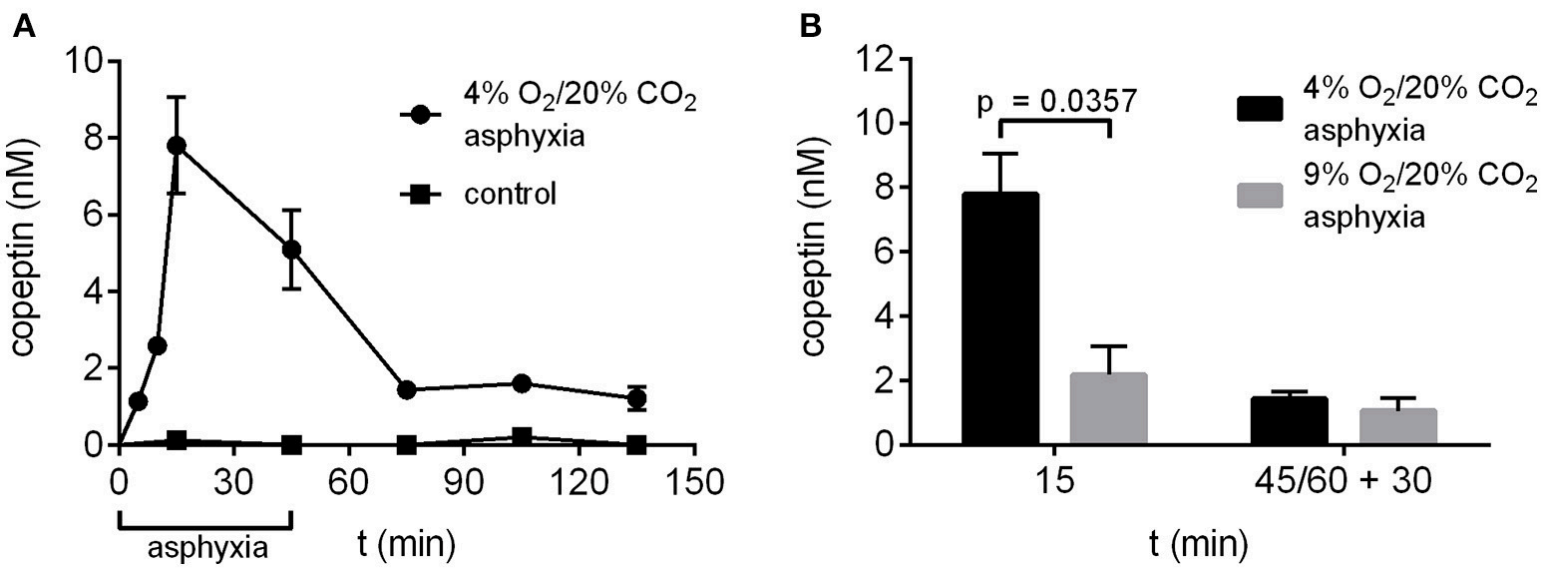
Copeptin concentrations in the P6 rat model of birth asphyxia. **(A)** Plasma copeptin levels in male P6 rats undergoing asphyxia and in controls breathing room air. Maximum plasma copeptin levels were observed after 15 min of asphyxia, after which copeptin levels steadily decreased until a plateau was reached at 30 min post-asphyxia. For asphyxia, *n* = 3 at 5 min of asphyxia, and 5–8 at all other time points. For controls, *n* = 3–4 at all time points. **(B)** Plasma copeptin levels were lower when rats were exposed to 9% O_2_/20% CO_2_ asphyxia compared to 4% O_2_/20% CO_2_ asphyxia. For 4% O_2_/20% CO_2_ asphyxia, *n* = 5 at 15 min and 7 at 45+30 min. For 9% O_2_/20% CO_2_ asphyxia, *n* = 3 at both time points. Copeptin concentrations were measured with the ELISA, and the data are shown as mean ± SEM. In **(B)** *p*-values from Mann–Whitney *U*-tests are shown when the difference was statistically significant.

The time-dependent changes in copeptin levels were studied by exposing P6 rat pups to 4% O_2_/20% CO_2_ asphyxia for a period of 45 min. The peak in mean copeptin concentration (7.8 ± 1.3 nM) was observed at 15 min after the start of asphyxia (Figure 2A), and by the end of asphyxia at 45 min, the mean copeptin level had decreased to 5.1 ± 1.0 nM, followed by a further decrease to 1.4 ± 0.2 nM at 30 min post-asphyxia. After that, the copeptin levels stayed relatively stable beyond 90 min post-asphyxia, indicating that a significant amount of copeptin release was still ongoing during the recovery period. In control animals breathing room air, copeptin concentrations remained close to 0 nM at all time points studied (Figure 2A). Thus, experimental asphyxia leads to enhanced AVP/copeptin secretion in rat pups in the 4% O_2_/20% CO_2_ asphyxia model.

In our previous work (Helmy et al., 2011, 2012) we have used an experimental paradigm where P6 rat pups were exposed to 9% O_2_/20% CO_2_ asphyxia for a period of 60 min. Therefore, we compared asphyxia induced copeptin release using the two O_2_ levels and application times. After 15 min of asphyxia the mean copeptin concentration was significantly lower in rat pups exposed to 9% O_2_/20% CO_2_ compared to 4% O_2_/20% CO_2_ (2.2 vs. 7.8 nM; Figure 2B). This was still the case at 30 min post-asphyxia (1.1 vs. 1.4 nM), but the difference was no longer statistically significant.

### Copeptin levels after intermittent asphyxia

**Figure 3:**
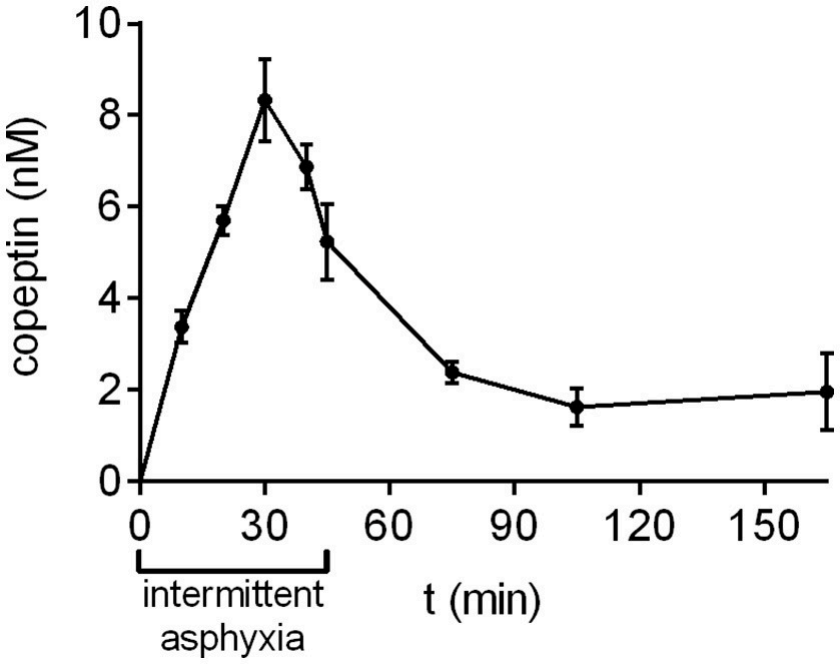
Copeptin concentrations during and after intermittent asphyxia. Maximum plasma copeptin concentrations were observed at 30 min following the onset of intermittent asphyxia, after which copeptin levels steadily decreased. *n* = 4–6 samples for all time points. Copeptin concentrations were measured with the AlphaLISA, and the mean ± SEM is shown for each time point.

During prolonged, complicated labor the oxygen levels experienced by the fetus are not steady, but fluctuate for example due to uterine contractions. In order to simulate this aspect of birth more accurately, we developed a model of intermittent asphyxia, where the oxygen level alternated between 9 and 5% every 5 min with the CO_2_ level kept at 20%. In this model, the highest copeptin concentrations were observed at 30 min after the start of asphyxia (8.3 ± 0.9 nM; Figure 3), followed by a decrease to 5.2 ± 0.8 nM at the end of asphyxia at 45 min. Thirty minutes after the end of asphyxia the mean copeptin concentration was 2.4 ± 0.2 nM. Thereafter, copeptin levels stayed elevated at around 2 nM beyond 120 min post-asphyxia (Figure 3).

### What is the trigger for copeptin release, hypoxia or hypercapnia?

**Figure 4:**
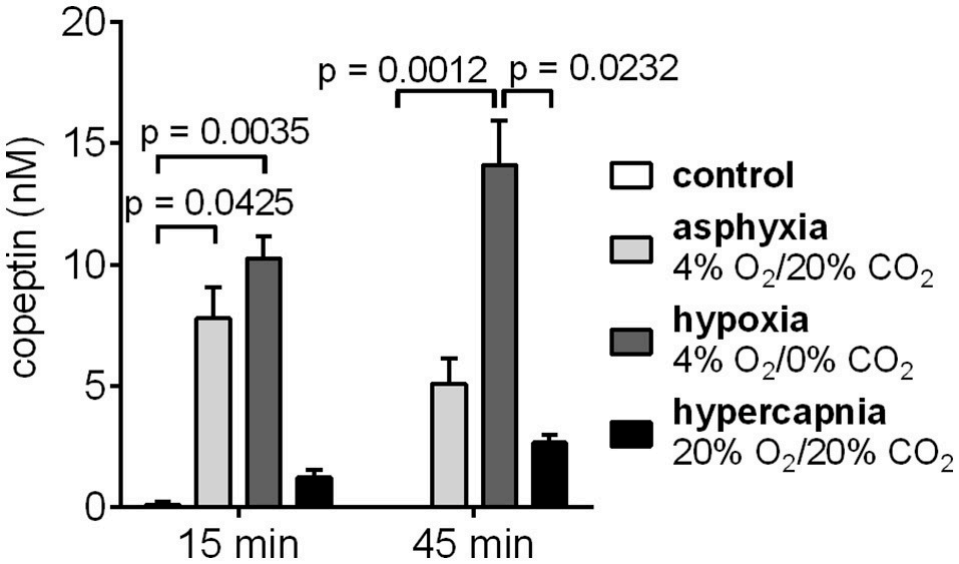
Copeptin blood levels in male P6 rats during 45 min exposures to 4% O_2_/20% CO_2_ asphyxia, 4% O_2_/0% CO_2_ hypoxia, and 20% O_2_/20% CO_2_ hypercapnia. The highest copeptin levels were observed after 45 min of hypoxia. *n* = 4–6 for all conditions, except for *n* = 3 for controls at 45 min. Copeptin concentrations were measured with the ELISA, and the mean ± SEM is shown for each condition. *p*-values from Kruskall–Wallis tests are shown where the difference was statistically significant.

The effects of hypoxia and hypercapnia, the two components of asphyxia, on copeptin release were studied separately by exposing the rat pups to either hypoxic or hypercapnic conditions for a period of 45 min. Hypoxia induced the highest increase in copeptin concentrations (Figure 4), with means of 10.3 and 14.1 nM at 15 and 45 min, respectively (compared to 7.8 and 5.1 nM for asphyxia, and 1.2 and 2.7 nM for hypercapnia).

### Decrease in pH and increase in negative base excess during experimental asphyxia

**Figure 5:**
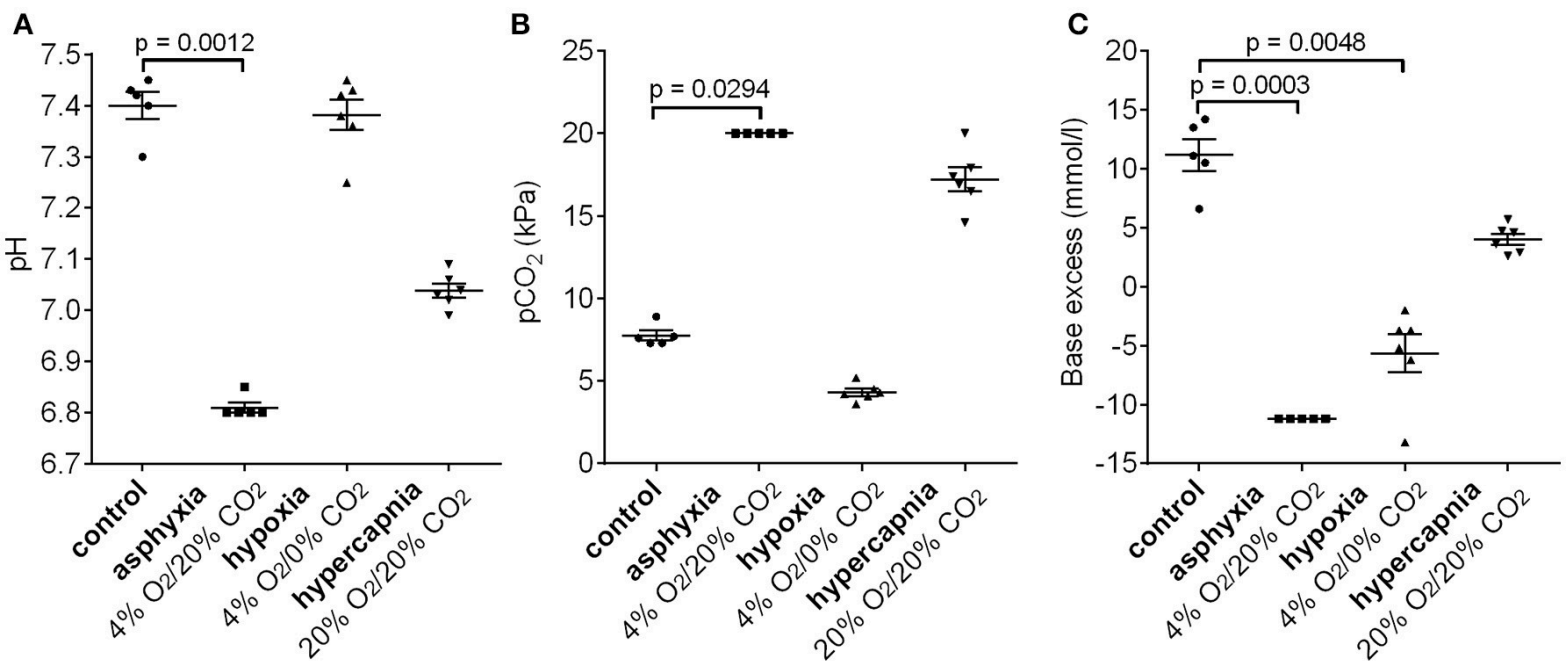
Changes in pH **(A)**, pCO_2_ **(B)**, and base excess **(C)** after 15 min of 4% O_2_/20% CO_2_ asphyxia, 4% O_2_/0% CO_2_ hypoxia, and 20% O_2_/20% CO_2_ hypercapnia. See section Materials and Methods for an explanation of the cut-off values in the 4% O_2_/20% CO_2_ asphyxia group. Means ± SEM are shown. *p*-values from Kruskall–Wallis tests are shown where the difference was statistically significant.

To examine whether the 4% O_2_/20% CO_2_ asphyxia model reproduces the standard diagnostic characteristics of birth asphyxia (Azzopardi et al., 2009; Schlapbach et al., 2011; Summanen et al., 2017), we measured the blood gas values in asphyxiated and control pups (Figure 5). After 15 min of asphyxia, the blood pH fell to levels of 6.8 or lower, and BE was below −11.2 mM. The pH and BE were significantly lower in asphyxiated compared to control pups, whereas the pCO_2_ levels were significantly higher in the asphyxiated animals compared to controls. Thus, our P6 model does not only reproduce the AVP/copeptin response to asphyxia, but it also demonstrates the presence of a significant negative BE.

Interestingly, a 15 min period of hypoxia did not alter the blood pH compared to control animals (Figure 5A). Moreover, the pCO_2_ levels were lower than in the controls, leading to the generation of a negative BE (mean −5.7 mM, Figure 5B). In contrast, 15 min of hypercapnia caused a decrease in blood pH, and an increase in pCO_2_ levels compared to control animals, but the BE remained positive (Figure 5).

## Discussion

AVP is an evolutionarily ancient signaling factor, which targets the V1a, V1b, and V2 receptors (Koshimizu et al., 2012) in various organ systems, including those that are known to be prone to the hypoxic challenge (in particular, the brain, kidneys, and liver) that takes place during both normal and complicated birth (Spoljaric et al., 2017). The fetal AVP surge occurring during normal vaginal birth was first described almost 50 years ago (Chard et al., 1971), and reports of the catecholamine surge followed a few years later (Lagercrantz and Bistoletti, 1977). AVP is derived from a larger precursor peptide, prepro-AVP, along with neurophysin II and the C-terminal 39-amino acid glycopeptide copeptin (de Bree and Burbach, 1998). During the last 10 years, copeptin has been used as an advantageous surrogate marker for AVP release (see section Introduction and references Morgenthaler et al., 2006, 2008). Several studies have reported an increase in serum copeptin levels following normal vaginal birth (Wellmann et al., 2010; Summanen et al., 2017), and, notably, the increase in copeptin levels is further accentuated by neonatal stress, such as birth asphyxia (Schlapbach et al., 2011; Summanen et al., 2017).

Here, we show for the first time that normal birth causes an increase in circulating levels of AVP/copeptin also in the rat. In plasma samples collected within 2 min after normal birth the mean copeptin concentration was 1.8 nM, compared to a mean of 0.9 nM in samples collected after cesarean section of the dam. The copeptin levels had decreased to baseline levels within 5–6 h after birth (Figure 1A). The cesarean section samples were collected at E21.5, which means that in some of the dams labor had probably started. This is likely why the copeptin concentrations in these samples are higher than those reported for human neonates after elective cesarean section. A 15 min period of experimental asphyxia was able to induce a second AVP/copeptin surge 5–6 h after normal birth (Figure 1B).

Moreover, we show that a rat model of birth asphyxia replicates the clinical findings of increased AVP/copeptin release in asphyxiated neonates. Sampling at several time-points allowed us to study the time-dependent changes in copeptin release during and after the asphyxic period. When pups were exposed to 4% O_2_/20% CO_2_ asphyxia for 45 min, plasma copeptin levels peaked already after 15 min of asphyxia (mean 7.8 nM), after which the levels steadily decreased. Considering the around 30 min half-life of copeptin in plasma (L'Abate et al., 2013), it is clear that the bulk of copeptin release in this model occurs at the very beginning of asphyxia, but that some release is still ongoing even 90 min after the end of asphyxia. In the clinic, the first sample available for biomarker analysis is from the umbilical cord, but the asphyxic period could have occurred at any point during labor. Therefore, based on these results, it is reasonable to assume that the AVP/copeptin levels in the neonate are even higher *in utero* than the extremely high values measured from umbilical cord blood. In fact, in our previous study we reported copeptin values of 2.3 nM in umbilical cord samples of human neonates diagnosed with birth asphyxia (Summanen et al., 2017), which is in the same order of magnitude as the 5.1 nM reported here for the end of asphyxia.

Notably, our present data show that the copeptin levels did not fall to the control value during the recovery period but remained at an elevated level, which indicates that sustained peripheral AVP release takes place for a lengthy period of time after the asphyxia. Thus, the various actions of AVP, including the adaptive ones described in the Introduction, do not end abruptly after the asphyxic insult. Moreover, it is important to note that the post-asphyxia levels of copeptin seen in the present model are analogous to those measured in neonatal blood samples after birth (Kelen et al., 2017). In our previous work on copeptin as a diagnostic and prognostic biomarker of HIE, the median copeptin concentration 1 week after birth in infants diagnosed with HIE was 17.8 pmol/l (Kelen et al., 2017), which is still above the normal range for healthy volunteers (Struck et al., 2005; Morgenthaler et al., 2006).

In human neonates, uterine contractions, which are accompanied by transient episodes of fetal hypoxia, are known to increase fetal copeptin levels as recently shown using an oxytocin-challenge test before elective cesarean section (Wellmann et al., 2016). Furthermore, copeptin levels strongly correlate with labor duration, and hence an increased number of contractions, and in particular the duration of the second stage of labor (Summanen et al., 2017). Therefore, we developed another paradigm (intermittent asphyxia), where rat pups were exposed to alternating 5 min periods of 9% O_2_/20% CO_2_ asphyxia and 5% O_2_/20% CO_2_ asphyxia. The pulses of more severe asphyxia combined with milder asphyxia were designed to mimic uterine contractions. In intermittent asphyxia copeptin levels peaked at 30 min of asphyxia (vs. 15 min as measured in the 4% O_2_/20% CO_2_ asphyxia model), and the concentrations remained elevated at ~2 nM beyond 120 min post-asphyxia.

Interestingly, the highest copeptin concentrations (mean 14.1 nM) were measured after 45 min of hypoxia. Hypercapnia alone resulted in much lower copeptin release compared to asphyxia. Therefore, it is the hypoxia component of asphyxia that is responsible for the increased AVP/copeptin release observed during asphyxia. This is in agreement with previous results from adult rats, where acute hypoxia resulted in significantly increased copeptin release (L'Abate et al., 2013; Ostergaard et al., 2014). Furthermore, it has been shown that acute hypoxia activates AVP-neurons in the PVN (King et al., 2012; Coldren et al., 2017). Importantly, there was no increase in copeptin concentrations in any of the control animals breathing room air. Therefore, the stress from the experimental paradigm, including maternal separation, was not sufficient to induce copeptin release in the 6-day old rat pups.

The standard diagnostic criteria for birth asphyxia usually include a fall in blood pH and a negative base excess. Typically, the criterion level of acidotic blood pH for birth asphyxia diagnosis is set between 7.0 and 7.1, with an additional and highly important criterion of a BE level in the range of −12 to −16 mmol/l (Azzopardi et al., 2009; Schlapbach et al., 2011; Kelen et al., 2017; Summanen et al., 2017). In our 4% O_2_/20% CO_2_ asphyxia model, the blood pH was <6.8 after 15 min of asphyxia, and the BE was <−11.2 mmol/l (see section Materials and Methods for an explanation of the cut-off values). Therefore, in addition to mimicking the neuroendocrine response of clinical birth asphyxia, our model also satisfies the standard acid-base diagnostic criteria of birth asphyxia. In our previous work we have used a model where 6-day old rat pups were exposed to 9% O_2_/20% CO_2_ asphyxia for 60 min (Helmy et al., 2011, 2012). However, in the 9% O_2_/20% CO_2_ asphyxia model the blood pH at the end of asphyxia was 7.25 (Helmy et al., 2011), which was significantly lower compared to controls but above the threshold for birth asphyxia diagnosis. This, coupled with the fact that copeptin release was significantly lower during 9% O_2_/20% CO_2_ asphyxia compared to 4% O_2_/20% CO_2_ asphyxia, indicates that an oxygen level of 9% is not sufficient to model clinical birth asphyxia in the 6-day old rat pup.

Animal models have been extensively used in studies of birth asphyxia (see section Introduction; and references Painter, 1995; Vannucci and Vannucci, 1997; Johnston et al., 2005; Mallard and Vexler, 2015) but, as far as we know, our present study is the first one to show that, in a clinically relevant rodent model of birth asphyxia as indicated by the relevant blood parameters, a massive surge of AVP takes place. This kind of a mechanism is likely to play an important role in “developmental plasticity” of an individual, thereby contributing to the allostatic, preadaptive mechanisms (McEwen, 1998) that help the individual to adjust to the process of birth itself, and also to the postnatal conditions experienced afterwards (Hanson and Gluckman, 2014). To conclude, the data presented here provide important mechanistic insights on the kinetics of AVP/copeptin release triggered by asphyxia: namely that most of the release occurs at the beginning of asphyxia; and the release continues after the asphyxia ends, which is in agreement with results from human neonates (Kelen et al., 2017). Notably, the data on both copeptin release and blood-gas parameters validate the current rodent model for preclinical work on birth asphyxia.

## Author contributions

MS carried out animal experiments, collected plasma samples, performed blood gas analyses, did the copeptin measurements, analyzed the data, and drafted the manuscript. SB carried out animal experiments, collected plasma samples, and was involved in writing the manuscript; JV was involved in conceptualizing and designing the work, and in writing the manuscript; KK is the project leader, designed the work, and drafted the paper together with MS; All authors have read and approved the final manuscript.

### Conflict of interest statement

The authors declare that the research was conducted in the absence of any commercial or financial relationships that could be construed as a potential conflict of interest.
