# Supplementary figures and images for: Surge of Peripheral Arginine Vasopressin in a Rat Model of Birth Asphyxia
Source: Front Cell Neurosci. 2018 Jan 19;12:2. doi: 10.3389/fncel.2018.00002 (PMC5780440; doi:10.3389/fncel.2018.00002)

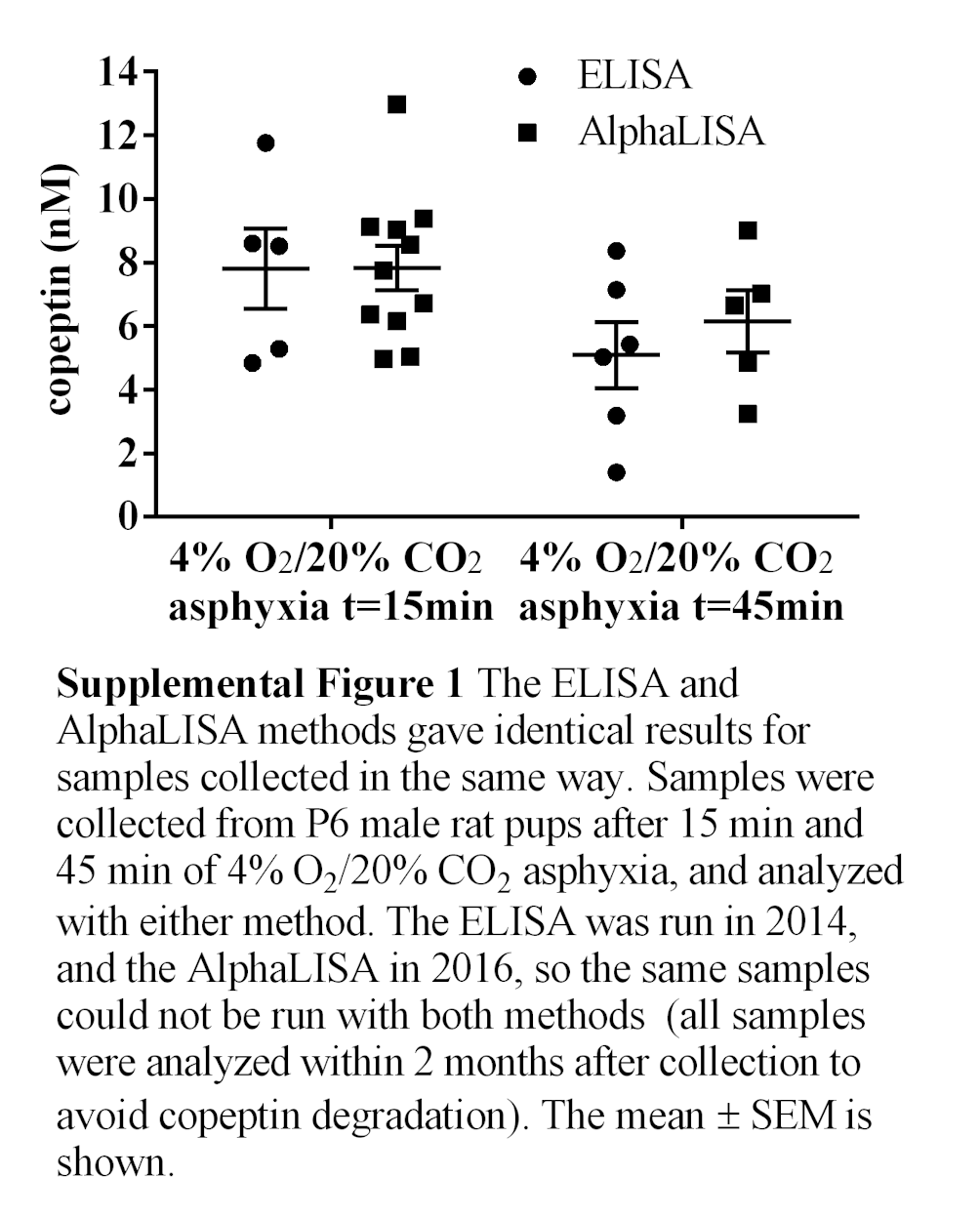

Supplement: Supplementary file 1 [file Image1.TIF]
